# Confirmation and Distribution of Tetrodotoxin for the First Time in Terrestrial Invertebrates: Two Terrestrial Flatworm Species (*Bipalium adventitium* and *Bipalium kewense*)

**DOI:** 10.1371/journal.pone.0100718

**Published:** 2014-06-25

**Authors:** Amber N. Stokes, Peter K. Ducey, Lorin Neuman-Lee, Charles T. Hanifin, Susannah S. French, Michael E. Pfrender, Edmund D. Brodie, Edmund D. Brodie Jr

**Affiliations:** 1 Department of Biology, California State University, Bakersfield, California, United States of America; 2 Biological Sciences Department, State University of New York, Cortland, New York, United States of America; 3 Department of Biology, Utah State University, Logan, Utah, United States of America; 4 Department of Biology, Utah State University – Uintah Basin, Vernal, Utah, United States of America; 5 Department of Biological Sciences, University of Notre Dame, Notre Dame, Indiana, United States of America; 6 Mountain Lake Biological Station and Department of Biology, University of Virginia, Charlottesville, Virginia, United States of America; United States Department of Agriculture, Beltsville Agricultural Research Center, United States of America

## Abstract

The potent neurotoxin tetrodotoxin (TTX) is known from a diverse array of taxa, but is unknown in terrestrial invertebrates. Tetrodotoxin is a low molecular weight compound that acts by blocking voltage-gated sodium channels, inducing paralysis. However, the origins and ecological functions of TTX in most taxa remain mysterious. Here, we show that TTX is present in two species of terrestrial flatworm (*Bipalium adventitium* and *Bipalium kewense*) using a competitive inhibition enzymatic immunoassay to quantify the toxin and high phase liquid chromatography to confirm the presence. We also investigated the distribution of TTX throughout the bodies of the flatworms and provide evidence suggesting that TTX is used during predation to subdue large prey items. We also show that the egg capsules of *B. adventitium* have TTX, indicating a further role in defense. These data suggest a potential route for TTX bioaccumulation in terrestrial systems.

## Introduction

Tetrodotoxin (TTX) is a low molecular weight neurotoxin that acts by blocking voltage-gated sodium channels in muscle and nerve tissues in many animal phyla, preventing the initiation and propagation of action potentials resulting in paralysis [1], [2]. In mammals, death often occurs in response to paralysis of the diaphragm and subsequent asphyxiation [3]. Tetrodotoxin is particularly interesting because it is found in a wide array of taxa ranging from bacteria to vertebrates, and the origins of the toxin are not understood [4]. Though TTX has been found in many marine invertebrate species including planarians, annelids, and molluscs, it has never been found in a terrestrial invertebrate.

*Bipalium adventitium* and *Bipalium kewense* are terrestrial flatworms invasive to the United States from southeastern Asia. Both species exhibit behaviors that indicate the possible use of a toxin to subdue large earthworm prey items [5], [6], [7], [8]. *Bipalium adventitium* are active hunters of earthworms that trail and attack earthworms 100 times their own mass [5], [9]. When the flatworm encounters earthworm prey, the flatworm crawls up the earthworm’s body towards the head. The flatworm then performs a behavior known as “capping”, in which it uses its head and body to cover the anterior end of the earthworm [5]. Capping has been found to significantly reduce the amount of movement and escape behavior exhibited by the earthworm [5]. The flatworm mouth, which is used during feeding and removal of waste, is located approximately one third to one half of the way down the ventral side of the body. Shortly after capping, the pharynx expands and attaches to the earthworm, releasing enzymes to externally digest the prey, and then ingests the meal. Earthworms will often struggle violently to escape once the flatworm’s pharynx is expanded and attached [5], [6], [7]. However, frequently following capping, and within a minute of expansion of the pharynx, the movements of the earthworm diminish and the earthworm seems to be paralyzed [5], [6], [7], suggesting that *Bipalium* may have a toxin that aids in subduing prey. Nevertheless, the presence of a toxin may also be utilized for defense against predators of *Bipalium*. In an effort to determine potential predators of *Bipalium*, non-TTX bearing salamanders were offered flatworms. In all cases where tongue contact or ingestion of the flatworm occurred salamanders rubbed their heads on the substrate; a typical response to TTX [5]. Given these observations, and the apparent paralysis in the earthworms, we hypothesized that the toxin in question may be TTX.

In this study we investigated the presence and distribution of TTX in *B. adventitium* and *B. kewense*. These two species have become relatively common throughout parts of the United States, and have the potential to greatly impact earthworm populations through predation [9]. Further, there is currently little known about predators of *Bipalium* in the United States, so it is important to understand their use of TTX if present. These two species of flatworm have the potential to greatly impact the health of native populations of both their own predators and prey. It is likely that TTX is used both defensively (against predators) and during predation (against prey) in *Bipalium* sp. However, if TTX were used primarily in a predatory function, it would likely be more concentrated in the head and mouth regions, whereas a defensive use typically is associated with a more even distribution throughout the body. We measured TTX levels using a Competitive Inhibition Enzymatic Immunoassay (CIEIA) technique [10], and confirmed using HPLC analysis. Distribution of TTX in flatworms was measured in three regions by segmenting worms into head, anterior body (from head to just beyond the mouth), and posterior body regions (remainder of body) (see supplementary materials). We also tested egg capsules of *B. adventitium* (*B. kewense* does not generally deposit egg capsules) for the presence of TTX. Toxic eggs provide further evidence for TTX as a defensive toxin in these flatworm species as seen in marine flatworms [11], blue-ringed octopus [12], and rough-skinned newts [13] among others.

## Materials and Methods

### Animal care/collection

*Bipalium kewense* were collected from Santa Clara County, CA (37°17′N, 121°43′W) and Harris County, TX (29°47′N, 95°18′W). *B. adventitium* were collected from Cortland County, NY (42°37′N, 76°04′W) and Schenectady County, NY (42°50′N, 74°03′W). No specific permissions were required for collection of these unprotected species at the collection sites. Individuals were shipped to Utah State University in Logan, UT, where they were housed prior to experimentation. Flatworms were housed individually in small (1-L) plastic containers with pinholes punched in the lids to promote airflow, and a damp paper towel for substrate. Flatworms were fed earthworms (*Eisenia fetida*, *Eisenia hortensis*, and *Lumbricus terrestris*) that were cut into pieces about two times the weight of the flatworm every other week. Containers were cleaned regularly with tap water. Between feedings the substrate was sprayed with tap water periodically to keep the environment moist. Containers were stored in larger dark plastic tubs to ensure protection from light.

### Extractions and TTX analysis

*Bipalium* individuals (N = 6 for each species) for whole flatworm analysis were weighed, placed in microcentrifuge tubes and frozen at −80°C. *Bipalium* individuals (N = 6 for each species) for segmented trials were cut into three portions using a scalpel. The head portion was cut just posterior to the head in the “neck region”. The anterior body portion of the flatworm was from the initial cut of the head posteriorly to the mouth. In cases where it was difficult to see the location of the mouth, a blunt probe was gently rubbed on the ventral side of the flatworm until some of the pharynx protruded. The posterior body region was the remainder of the flatworm beyond the mouth. All three segments were individually weighed, placed in labeled microcentrifuge tubes, and frozen at −80°C. *Bipalium adventitium* that had arrived in the lab produced egg capsules shortly after arrival (N = 8). These capsules were also frozen at −80°C and weighed prior to extraction for TTX quantification.

Tetrodotoxin was extracted from flatworms using the methods of Hanifin et al. [14]. Flatworms were homogenized in either 600 or 800 µl of 0.1 M acetic acid and boiled for five minutes. Each sample was then centrifuged at 13,000 RPMs for 20 minutes. The supernatant was then pipetted into centrifugal filter tubes and centrifuged in the same manner as before. All samples were stored at −80°C for quantification. Quantification of TTX was done using a Competitive Inhibition Enzymatic Immunoassay as in Stokes et al. [10]. Standards were prepared from TTX-citrate purchased from Abcam, and diluted in 0.1 M acetic acid to the linear range of the standard curve, which was between 10 and 500 ng/mL. Values less than 10 ng/mL are referred to as below detectable limits (BDL) and considered as zero in our analyses. Samples were not diluted. The average coefficient of variation on each plate was between 4.05–4.51%.

Qualitative analysis and confirmation of the presence of TTX was performed using High Performance Liquid Chromatopgraphy (HPLC) with fluorescence detection. Protocols were largely similar to previously published methods [15], [16], [17]. Separation of TTX and TTX analogs was performed with mobile phase that consisted of 50 mM ammonium acetate and 60 mM ammonium heptafluorobutyrate buffers (pH 5.0) with 1% acetonitrile and a Synergi 4 µ Hydro-RP 80A column (4.6×250 mm, Phenomenex, USA) on a Beckman 126 pump system. Samples were injected using a Beckman 508 autosampler. Post column derivitization was achieved using a Pickering CRX 400 post column reactor set at 115°C in 5N NaOH. Fluorescence was measured using a JASCO 1520 detector (excitation wavelength: 365 nM, emission wavelength: 510 nm). Data acquisition and chromatographic analysis was performed using 32 K System Gold software (version 8.0) and an SS420x A/D convertor (Agilent Technologies). Flatworm extracts were compared to commercially sourced authentic TTX standards (see above) to confirm the presence of authentic TTX.

### Statistical analyses

For both whole flatworm and segmented flatworm samples we looked at the overall amount of TTX in the sample (concentration), as well as the amount of TTX in the sample relative to body weight of the flatworm or segment. Whole flatworm data did not meet the assumptions of normality and heterogeneity, so were compared using non-parametric Wilcoxon Kruskal Wallis tests using JMP version 10 (SAS Institute). Segmented flatworm samples were analyzed as a split plot design with each individual flatworm as the block, species as the whole plot factor, and segment as the subplot factor. Data for segmented flatworms did not meet the assumptions of normality and heterogeneity. Concentration data were square-root transformed, and data adjusted for body weight were log transformed to better meet these assumptions. Analyses of segmented flatworms were performed using PROC GLIMMIX in SAS version 9.3 (SAS Institute).

## Results

**Figure 1:**
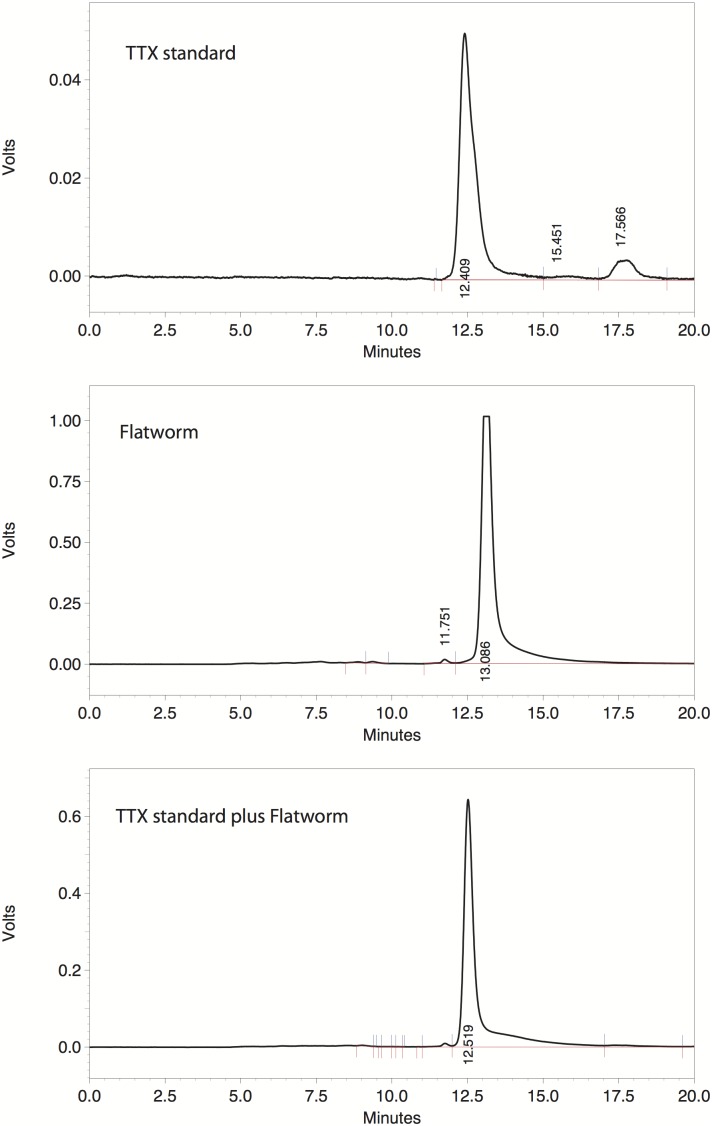
HPLC diagram confirming the presence of TTX in flatworm tissue. Elution times and TTX profiles of an authentic TTX standard (Top), *Bipalium adventitium* (middle), and *B*. *adventitium* co-injected with a TTX standard (0.0005 mg/ml). The presence of single peak in the flatworm and co-injected sample confirm that the TTX like toxin present in this species is authentic TTX.

We confirmed the presence of TTX in these species using HPLC analysis (Fig. 1). We compared the peaks from *B. adventitium* to that of a 0.0005 mg/mL TTX standard, and the flatworm sample co-injected with the same TTX standard. The single peak in the flatworm and in the co-injected sample indicates that this toxin is TTX and that TTX itself is the primary enantiomer present in these species.

**Figure 2:**
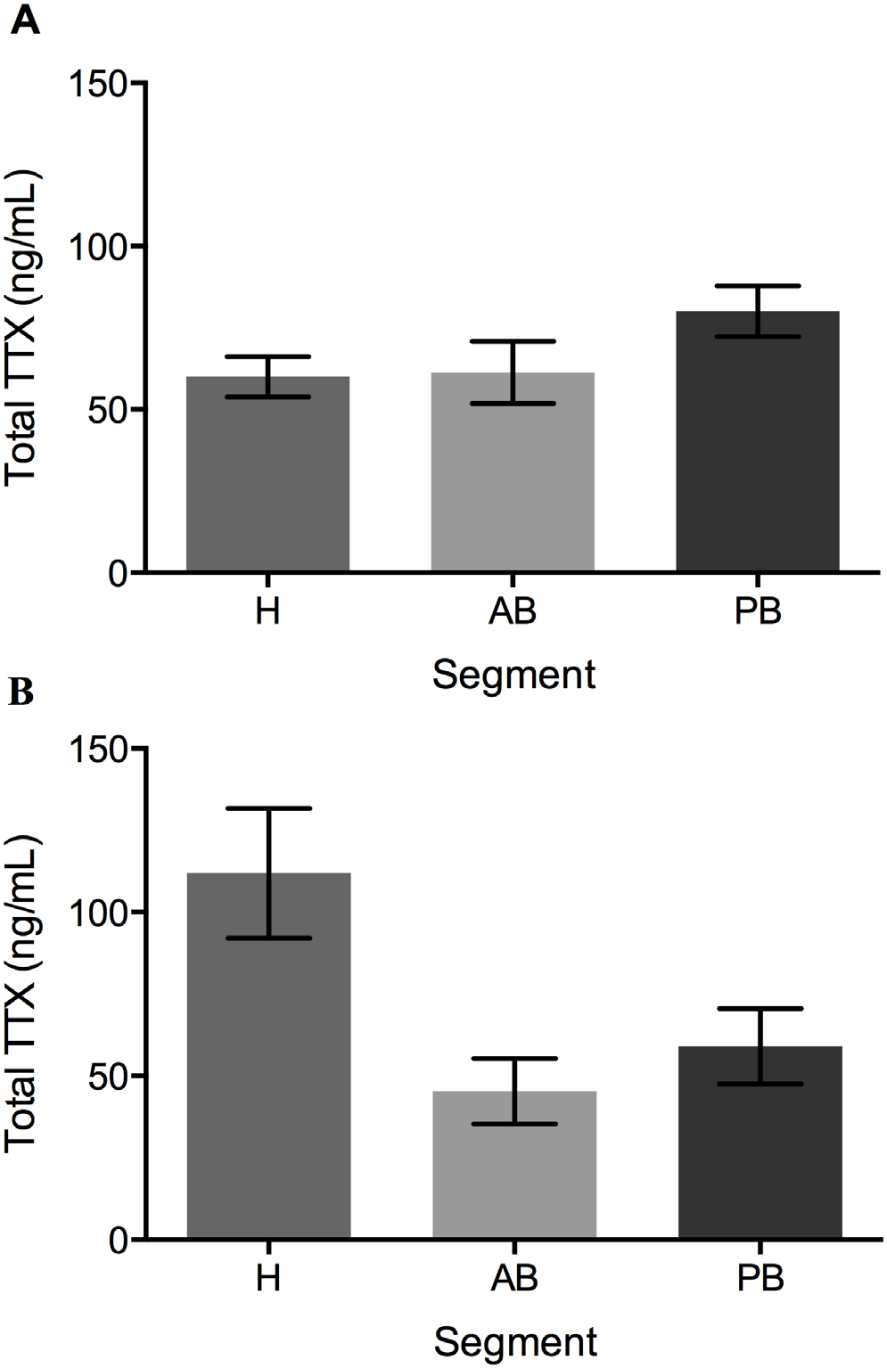
Total TTX (ng/mL) for each segment for (a) *Bipalium adventitium* and (b) *Bipalium kewense*. Segments are denoted as H for head, AB for anterior body, and PB for posterior body.

**Figure 3:**
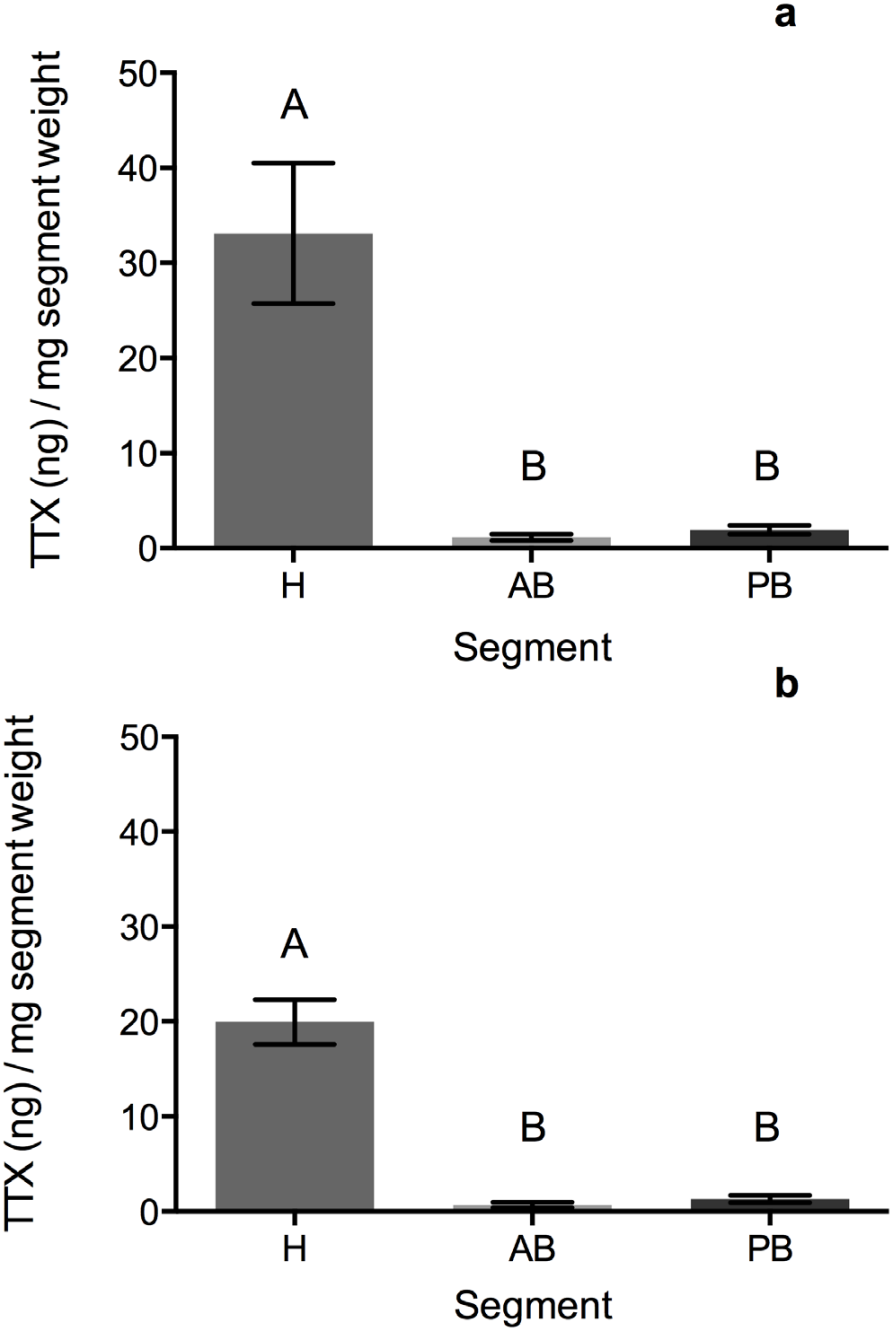
The amount of TTX (ng) adjusted segment weight (mg) for (a) *Bipalium adventitium* and (b) *Bipalium kewense*. Segments are denoted as H for head, AB for anterior body, and PB for posterior body. Letters above bars indicate significant differences between segments.

Both *Bipalium kewense* and *B. adventitium* exhibited TTX (Fig. 1, 2, and 3). Total TTX did not differ between the two species (df = 1, P = 0.4712). The mean amount of total TTX in each sample for *B. adventitium* and *B. kewense* was 40.10 ng/mL TTX (SEM = 14.218, Range = BDL–81.42 ng/mL) and 62.03 ng/mL TTX (SEM = 5.13, Range = 50.25–82.71 ng/mL), respectively. Likewise, relative to mass the two species did not differ with a mean of 4.64 ng/mg TTX (SEM = 3.26, Range = BDL–19.89 ng/mg) for whole *B. adventitium*, and a mean of 3.72 ng/mg TTX (SEM = 1.22, Range = 0.73–8.27 ng/mg) for whole *B. kewense*.

Comparisons of segments indicated no differences between total TTX in the two species (F = 0.00, P = 0.9926, Fig. 2, Table 1), or between the segments (F = 2.76, P = 0.0875). There was, however, a significant interaction between species and segment (F = 3.94, P = 0.0361), due to slight differences in the distribution of the toxin. Total TTX was greater in the anterior and posterior body regions for *B. adventitium* (Fig. 2a, Table 1), but was greater in the head of *B. kewense* (Fig. 2b). Relative to mass, segmented *B. adventitium* had significantly more TTX than did segmented *B. kewense* (F = 4.95, df = 1, P = 0.0364). There were also significant differences in the amount of TTX in each segment for both species (F = 89.37, df = 2, P<0.0001, Fig. 3), with the head having significantly more TTX than the anterior body (t = 11.57, P<0.0001) and the posterior body (t = −8.23, P<0.0001). The anterior and posterior body segments have similar levels of TTX relative to mass (t = 1.58, P = 0.2761). Unlike total TTX, there were no significant interactions between species and segment relative to mass (F = 0.30, df = 2, P = 0.7444), as both species had similar patterns of TTX distribution in the body regions. *Bipalium adventitium* eggs were also found to have TTX, with a mean of 89.73 ng of TTX total (SEM = 7.14, Range = 49.18–110.30) and an average of 10.56 ng TTX/mg weight (SEM = 2.62, Range = 2.06–22.33).

**Table 1:** The amount of TTX (ng) adjusted for weight of body segment (mg) for *Bipalium adventitium* and *Bipalium kewense*.

	*Bipalium adventitium*			*Bipalium kewense*		
Body Region	Mean	Range	SEM	Mean	Range	SEM
Head	33.11	14.99–62.66	7.40	19.96	12.28–26.98	2.34
Anterior body	1.15	0.23–2.31	0.33	0.69	0.08–2.10	0.30
Posterior Body	1.94	0.65–1.94	0.47	1.33	0.06–2.34	0.37

## Discussion

Although, TTX is best known as a defensive compound in vertebrates, there are many invertebrate groups that have TTX including red calcareous algae, dinoflagellates, bacteria, horseshoe crabs, blue-ringed octopuses, and multiple species of gastropods [4]. Notably, several groups of worms possess TTX including annelids and flatworms (*Planocera* species) [4], [18]. Though this taxonomic distribution is wide, this study is the first known to document the presence of TTX in any terrestrial invertebrate species.

Marine planocerid flatworms have been documented using TTX in predation to subdue mobile prey items [18]. Additionally, previous studies using *Bipalium* documented that earthworms displayed partial paralysis and were likely subdued with a toxin following attack [5], [6], [7]. Our study shows that there is a large amount of TTX relative to mass found in the heads of these flatworms. The heads of *Bipalium kewense* are highly innervated and possess ciliated sensory organs [19], therefore, it is possible that some release of TTX occurs from the head during capping. However, the importance of TTX release from the mouth during feeding cannot be ruled out at this time. It should be noted that we did not document the actual release of TTX or confirm the role of TTX in the paralysis observed in earthworms in this study. Further study is needed to confirm the role of TTX in predation.

These observations are not sufficient to rule out an additional defensive role of TTX in these species. Tetrodotoxin is evenly distributed throughout the body, indicating a possible role in defense. The feeding trials conducted by Ducey et al. [5] using potential salamander and snake predators of *B. adventitium* indicate that predation by these species may be deterred in two ways. First, none of the predators readily identified *Bipalium* as a prey item, with 90% of the salamanders, and all of the snakes failing to strike at the flatworms. Secondly, all but two of the flatworms that were struck were rejected. Those that rejected the flatworms rubbed the sides of their heads on the substrate following contact with the flatworm, which is a common response to contact with TTX laden prey [3]. When habituated salamanders were offered *B. adventitium* with forceps, all of the salamanders struck, and only three of nine rejected the flatworms [5]. Again, all salamanders responded by rubbing their heads on the substrate and gaping their mouths at least once. Though these trials with habituated salamanders indicate that salamanders can eat *B. adventitium* without dying, it is unknown if future offerings of *Bipalium* would be rejected. This indicates that TTX functions well as an antipredator mechanism for *B. adventitium*, and likely equally well for *B. kewense*.

A defensive role for TTX in these flatworms is further supported by the presence and apparent maternal investment of TTX in the egg capsules of *Bipalium adventitium.* This type of investment has been well documented in vertebrate species that have TTX such as pufferfish [20], [21] and the rough-skinned newt, *Taricha granulosa* [13], [22]. The marine flatworm *Planocera multitentaculata*, also invest TTX into their eggs, and can lay eggs that are 2–50 times more toxic than the parent [11]. Our study showed that *B. adventitium* eggs contained approximately three times the amount of TTX on average (89.73 ng TTX) as the average whole flatworm sample (27.89 ng TTX). However, we also show that there is variation in the levels of TTX, and many of those sampled had more TTX total than did the eggs. Each *B. adventitium* egg capsule contains between one and six flatworms, with an average of approximately three flatworms per capsule [23]. Egg capsules sit unguarded in the soil for approximately three weeks prior to hatching. Therefore, a defensive toxin would be helpful in warding off opportunistic predators. It is unknown whether or not newly hatched flatworms also have TTX or if the toxin is contained solely in the egg capsule.

Currently, we have little conclusive knowledge regarding production or acquisition of TTX in the tissues of organisms. In marine systems the common view is that bacterial species produce TTX, which is then sequestered in the tissues of other organisms that ingest the bacteria [24]. Puffer fish often are the example of this hypothesis, as they have little to no TTX when not fed a TTX-bearing diet [25]. However, even in puffer fish it is unclear where exactly the TTX came from as the production of TTX by bacterial species is still questionable [26], and this hypothesis has not held up well in regards to terrestrial species [14], [22]. Our findings provide a potential avenue for TTX to move up the food chain in terrestrial organisms.

## Supporting Information

Table S1 Raw data for TTX levels and distribution in Bipalium adventitium and Bipalium kewense.
(XLSX)
